# Preformulation Studies for Generic Omeprazole Magnesium Enteric Coated Tablets

**DOI:** 10.1155/2015/307032

**Published:** 2015-01-28

**Authors:** C. O. Migoha, M. Ratansi, E. Kaale, G. Kagashe

**Affiliations:** ^1^Tanzania Food and Drugs Authority, P.O. Box 77150, Dar es Salaam, Tanzania; ^2^Pharm R&D Lab, School of Pharmacy, Muhimbili University of Health and Allied Sciences, P.O. Box 65013, Dar es Salaam, Tanzania; ^3^Department of Pharmaceutics, School of Pharmacy, Muhimbili University of Health and Allied Sciences, P.O. Box 65013, Dar es Salaam, Tanzania

## Abstract

Preformulation is an important step in the rational formulation of an active pharmaceutical ingredient (API). Micromeritics properties: bulk density (BD) and tapped density (TD), compressibility index (Carr's index), Hauser's ratio (H), and sieve analysis were performed in order to determine the best excipients to be used in the formulation development of omeprazole magnesium enteric coated tablets. Results show that omeprazole magnesium has fair flow and compressibility properties (BD 0.4 g/mL, TD 0.485 g/mL, Carr's index 17.5%, Hauser's ratio 1.2, and sieve analysis time 5 minutes). There were no significant drug excipient interactions except change in colour in all three conditions in the mixture of omeprazole and aerosil 200. Moisture content loss on drying in all three conditions was not constant and the changes were attributed to surrounding environment during the test time. Changes in the absorption spectra were noted in the mixture of omeprazole and water aerosil only in the visible region of 350–2500 nm. Omeprazole magnesium alone and with all excipients showed no significant changes in omeprazole concentration for a 30-day period. Omeprazole magnesium formulation complies with USP standards with regards to the fineness, flowability, and compressibility of which other excipients can be used in the formulation.

## 1. Introduction

**Figure 1:**
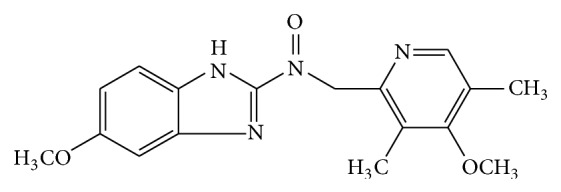
Structure of omeprazole.

Preformulation is the first step in the rational formulation of an active pharmaceutical ingredient (API). It is an investigation of the physicochemical properties of the drug substance, alone and in combination with excipients [1]. Omeprazole, 5-methoxy-2(((4-methoxy-3,5-dimethyl-2-pyridinyl)methyl)sulfinyl)-1H-benzimidazole, is a potent inhibitor of gastric acid secretion [2]; see Figure 1. It is among proton pump inhibitors (PPI) and shows powerful inhibitory action against secretion of gastric juice and is used in treatment of duodenal and gastric ulcers, erosive or ulcerative gastroesophageal reflux disease (GERD), and pathological hypersecretory conditions (Zollinger-Ellison) [2]. However, omeprazole is susceptible to degradation/transformation in acid reacting and neural media [3].

The in vitro degradation of omeprazole is catalyzed by acidic environment and is stabilized in mixtures in alkaline environment. Moisture and organic solvents also affect the stability of omeprazole [4]. From omeprazole stability studies data, it is obvious that an oral dosage form must be protected from contact with gastric juice in order to reach the small intestine without degradation [1, 3–5].

Therefore exposure of omeprazole magnesium to the acid condition of the stomach would lead to significant degradation of the drug and hence reduce bioavailability [6]. A pharmaceutical dosage form with properties of protecting omeprazole from contact with gastric acid must be developed, which is core tablet. The core must be enteric coated. The core developed must be alkaline in nature as most of available acidic coating materials such as phthalate-phthalic acid esters or acrylate-acrylic acid copolymers (KILLOCOATE MAE) will not favor stability of omeprazole [6, 7].

The aim of the present work was to perform preformulation studies to inform development of omeprazole enteric coated tablets for the purpose of determining the physical chemical characteristics of omeprazole with possible interactions with excipients.

## 2. Experimental

### 2.1. Equipment

Bulk density apparatus (IPA Flowmatics Pvt., Ltd., Bangalore, India), graduated cylinder (Fisher Scientific, Germany), sieve analyzer (Endecott's, Germany), glass bottles (Fisher Scientific, Germany), stability chambers (Thermolab Scientific Pvt., Ltd., Vasai, India), hot oven, near infrared absorption spectroscopy (Advanced System Development (ASD) Inc., NIR Systems, Boulder, Colorado, USA), HPLC (Shimadzu, Japan), and fast moisture tester (Guoming, China).

### 2.2. Materials

Omeprazole magnesium (Metrochem API Private Limited, Hyderabad, India), sodium lauryl sulphate (LOBA Chemie Pvt. Ltd., Mumbai, India), lactose (OXFORD Laboratories, Mumbai, India), Avicel PH 102 (Shandong Liaocheng Ehua medicine Co. Ltd., Shandong, China), maize starch (OXFORD Laboratories, Mumbai, India), water aerosil 200 (Shandong Liaocheng Ehua medicine Co. Ltd., Shandong, China), and magnesium stearate (Huzhou Zhanwang Pharmaceutical Co. Ltd., Huzhou, China) were used. Other reagents and solvents were procured commercially in local market and were of pharmaceutical and analytical grade.

### 2.3. Procedures

#### 2.3.1. Preformulation Studies

*(1) Micromeritics Properties of API*

*(A) Bulk Density and Tapped Density*. Both bulk density (BD) and tapped density (TD) including compressibility index were determined as prescribed in USP30NF35. The compressibility index of the powder blend was determined by Carr's index. This can be used to predict flow properties based on density measurement [6, 8]. The formula for Carr's index is shown below:

(1) $$\begin{array}{c} \text{Carr's}\;\text{index}\;\left(\%\right)=\frac{\text{Tapped}\;\text{density}-\text{Bulk}\;\text{density}}{\text{Tapped}\;\text{density}}*100. \end{array}$$

*(B) Hausner's Ratio (H).* This expresses the flow properties of the powder and is measured by the ratio of tapped density (TD) to bulk density (BD) [6, 8]. It is calculated from the following equation:

(2) $$\begin{array}{c} \text{Hauser}\;\text{ratio}=\frac{\text{TD}}{\text{BD}}. \end{array}$$

Powder blend was evaluated for bulk density and tapped density, compressibility index, and Hausner's ratio as described above. Additionally the assay was performed as per monograph for determination of omeprazole magnesium USP30NF35 [3].

**Figure 2:**
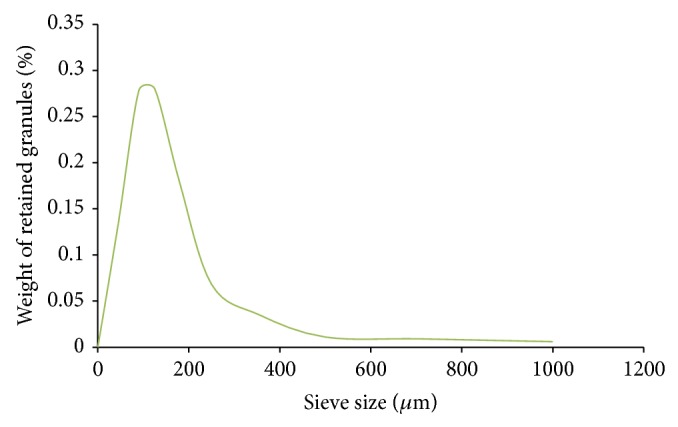
Sieve analysis cumulative weight of retained granules (%) versus sieve size (*μ*m) curve.

*(C) Sieve Analysis*. The main aim of sieve analysis was to determine the distribution of different size of drug particles present [8]. A series of sieves was arranged in the order of their deceasing pore diameter, that is, sieves numbers 0, 45, 90, 125, 180, 250, 355, 500, 710, and 1000. About 100 grams of drug was weighed accurately and transferred to sieve 1000 which was kept on the top. The sieves were shaken for about 5 minutes. Then the drug retained on each of the sieves was taken, weighted separately, and expressed in terms of percentage. The limit is expected as in Table 1 [8]. The results of sieve analysis are shown in Figure 2.

#### 2.3.2. Drug Excipients Compatibility Studies

Drug excipient compatibility studies were carried out by mixing drug with potential excipient. The ratio of formulation excipients to active substances was maintained at a ratio of 1 : 1. The mixtures were filled in closed vials and placed in stability chambers in the conditions as prescribed in US Pharmacopeia 30NF25 [4].  Table 2 depicts the composition of core tablets.

The preparation of samples for compatibility studies considered the amount of active ingredients and excipients as described in US Pharmacopeia 30NF25. The sample of the mixtures was mixed with API and excipients as prescribed in the study proposal. The set of samples were mixed well to ensure homogenicity.

The possible interactions between omeprazole magnesium and excipients were evaluated by examining the pure drug and drug excipient powder mixtures which were stored under conditions as depicted in Table 1 for a period of 90 days. The following parameters were assessed: appearance and colour, moisture content, near infrared (NIR), and assay by high performance liquid chromatography (HPLC) for omeprazole and related substances. The frequency of sampling was days 0, 3, 7, 30, 60, and 90.

The powder was scanned using NIR spectrophotometer. The procedure was started by cleaning the working area and assured that there was no dust. The device was switched on (Muglight and the laboratory spec.). The device was calibrated and the base line for omeprazole powder and mixture for day zero were recorded. The samples of the powder were filled in the vials and sample count was set to average of 30-sample count.

## 3. Results and Discussion

### 3.1. Micromeritics Properties

Results of micromeritics studies as presented in Table 3 showed that Hauser's ratio was 1.2 and Carr's index was 17.5% which indicates that omeprazole magnesium has fair compressibility and flowability properties, respectively. Therefore it is important to improve flow and compressibility property [4].

The results of sieve analysis show that omeprazole magnesium has a *D*_50_ value of 100 *μ*m which is within the range of 90 to 125 *μ*m and it indicates that omeprazole magnesium powder is moderately fine [4].

The results of Hauser ratio and compressibility index obtained have shown that flowability of omeprazole magnesium was fair. This will help in selection and determination of the optimal excipients and amount of the excipients to be used. That is, it is recommended that about 0.07% of magnesium stearate should be used to improve the flowability of the powder during formulation.

The flowability and compressibility of omeprazole magnesium powder indicate that the powder is suitable for both direct compression and wet granulation method depending on the other excipients used with their respective amounts that could be used instead.

Physical observation during drug excipient compatibility studies and physical observation indicates no significant drug-excipient interaction was observed except for the mixture of omeprazole and aerosil 200 which show change in colour in all three conditions. Therefore from the study it was concluded that the omeprazole magnesium and other excipients were compatible with each other as results are depicted in Table 4.

### 3.2. Moisture Content

The values of moisture content obtained by using loss on drying method indicate that the amount of moisture present in all the samples subjected to all three different storage conditions was not constant and the variation did not follow any specific trend as days progressed.

The change was probably due to the surrounding environment on that specific day and time when the samples were being analyzed for moisture content determination. Thus variation was observed in the moisture content but did not follow any trend with progression of the days.  Figure 11 indicates the comparison of moisture content in samples at different conditions of A, B, and C (Table 5).

### 3.3. Near Infrared Absorption Spectra

**Figure 3:**
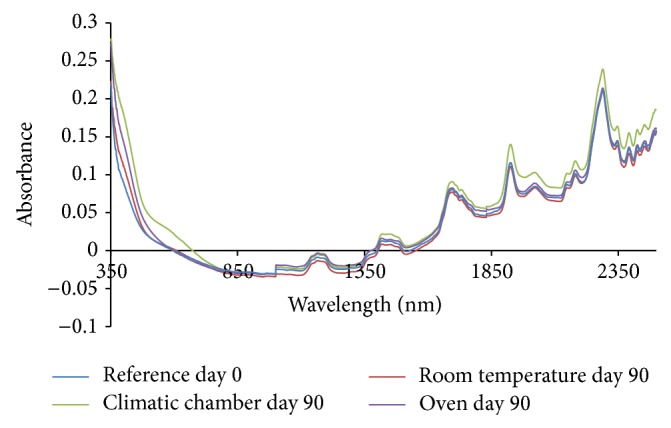
Absorption NIR spectra for omeprazole magnesium alone.

**Figure 4:**
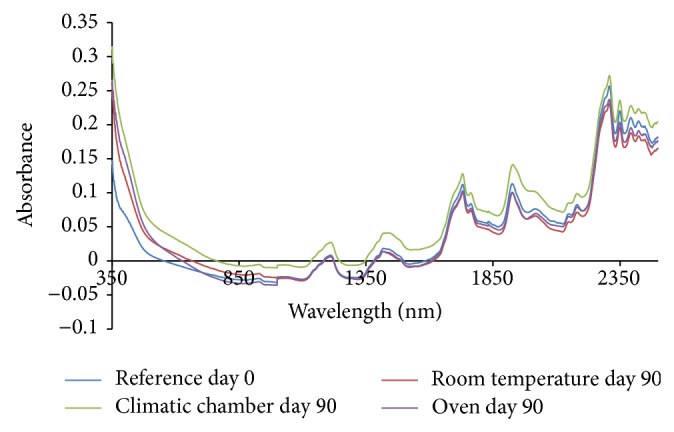
Absorption NIR spectra of omeprazole magnesium with sodium lauryl sulphate.

**Figure 5:**
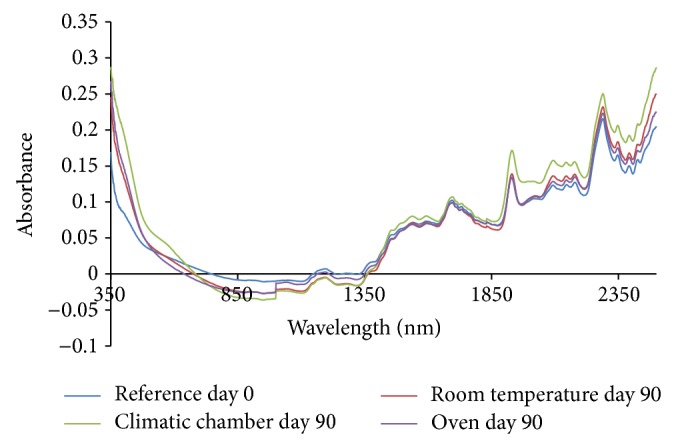
Absorption NIR spectra of omeprazole magnesium with lactose.

**Figure 6:**
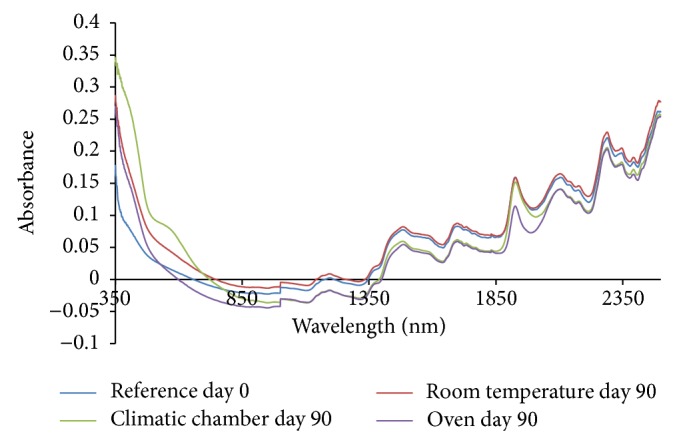
Absorption NIR spectra of omeprazole magnesium with Avicel PH 101.

**Figure 7:**
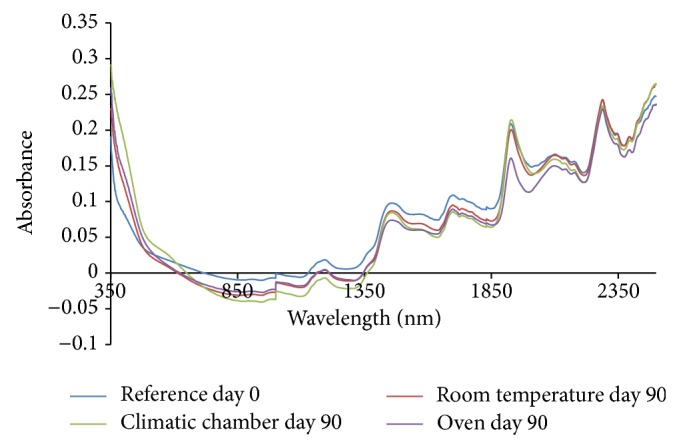
Absorption NIR spectra for omeprazole Mg with starch.

**Figure 8:**
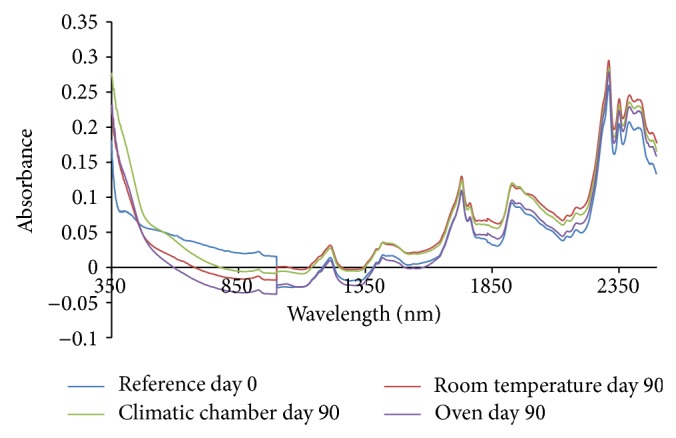
Absorption NIR spectra of omeprazole Mg with magnesium stearate.

**Figure 9:**
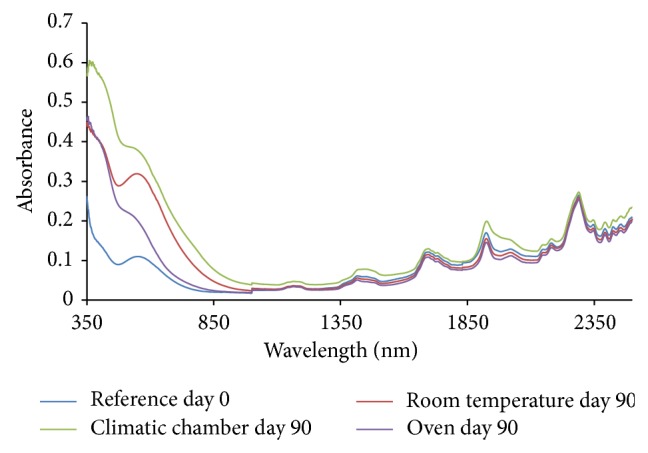
Absorption NIR spectra for omeprazole Mg with aerosil 200.

From the absorption spectra (omeprazole Mg alone, omeprazole Mg: sodium lauryl sulphate, omeprazole Mg: lactose, omeprazole Mg: Avicel PH 101, omeprazole Mg: starch, and omeprazole Mg: magnesium stearate) shown in Figures 3, 4, 5, 6, 7, 8, and 9, respectively, it can be observed that the spectra overlap each other and the peaks and troughs fall together with the reference spectra at different wavelengths of samples subjected to all three conditions throughout the 90-day period. There is no significant change in the nature of the absorption patterns and the minor changes observed in the spectra are a result of moisture variation in the samples.

By visual inspection of the spectra there is no significant change in the spectra from wavelength of 350–2500 nm which includes the visible region (350–700 nm) and near infrared region (800–2500 nm) but the important near infrared ranges from 1100 to 2500 nm as strong bands (1100–2500 nm) are related to overtone and combinations of fundamental vibrations of OH, NH, and CH which are present in omeprazole molecules.

The reference spectra at day zero were within and in between the other spectra showing there was no change in omeprazole Mg alone and with the excipients when subjected to different conditions for a period of 90 days.

There was no appearance change or colour change observed in these samples from day 0 to day 90 and all of them remained white thoroughly and it is verified from the spectra that there was no nature of change in absorption spectra even in the visible region from 350 to 800 nm; thus no colour change was observed in the samples.

**Figure 10:**
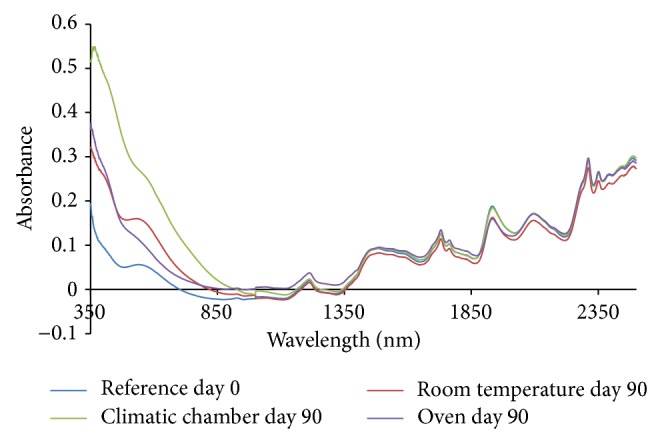
Absorption NIR spectra for omeprazole magnesium with all potential excipients.

**Figure 11:**
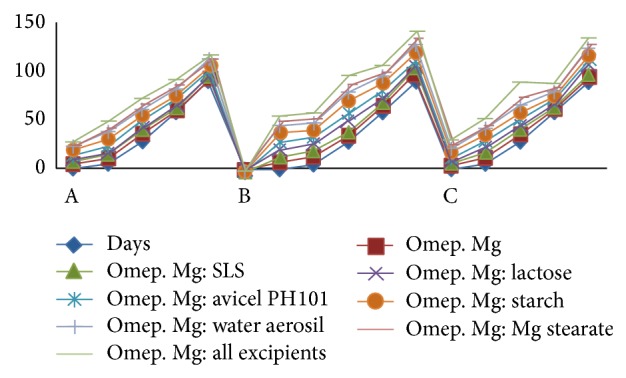
Comparison of moisture content in samples at different conditions A, B, and C.

From the absorption spectra of omeprazole magnesium with aerosil 200, that is, Figure 10, there is a change in the nature and absorption of spectra from 350 to 1000 nm which was mainly the visible region of the spectrum; hence colour change is observed in the samples as it is shown in the results of appearance.

The spectra of sample kept in the climatic chamber have deviated most in terms of absorption from day zero (reference spectrum) and that is why most of the colour change was observed in these samples, that is, white > dark purple > dark brown during the 90-day period.

The colour change was also observed in the sample kept in the oven from white > dark purple > light purple > light brown and this is verified by the change of absorption spectra in the visible region during the 90-day period.

Colour change was observed even in samples kept under room temperature from white > dark purple > purple and can be followed with the changes observed in the absorption spectra in the visible region.

For the sample kept in the climatic chamber and oven, there is a change in nature of spectra observed at wavelength of about 500–550 nm; these could be due to heating of the samples, that is, 40°C in climatic chamber and 50°C in oven. This change of nature was not observed in samples kept in the room temperature. The change is due to heating in both climatic chamber and oven.

Colour changes were observed in the samples subjected to all three conditions corresponding to changes in the absorption spectra in the visible region; however no major changes were observed in the important near infrared region (1100–2500 nm). The colour of the samples was changing, that is, physical changes, but there is no change in omeprazole structure as it can be observed that the spectra overlap each other and the peaks and troughs fall together in the important near infrared region throughout the 90-day period. The colour change was due to the reaction between magnesium and aerosil 200.

The absorption spectra for mixture of omeprazole magnesium with all potential excipients were used for the compatibility studies; changes in absorption spectra were seen only in the visible region (350–800 nm) in all three different conditions which also led to the change of colour in the samples as days progressed from day 0 to day 90 and this colour change is due to aerosil 200 excipient which reacted with magnesium part of omeprazole magnesium to form magnesium silicide which is purple in colour.

The absorption spectra in the near infrared region overlap each other and the peaks and troughs fall together. The reference spectrum (day 0 spectrum) was found in between other spectra in the near infrared region. This shows that there is no interaction between omeprazole and excipients. This shows that aerosil 200 is incompatible with omeprazole magnesium and should be avoided in the formulation phase.

### 3.4. Assay

The assay results in Table 6 showed that there were no significant changes in the concentration of omeprazole magnesium from day 0 to day 90 and this indicates that omeprazole magnesium is stable when subjected to conditions of different temperature and humidity. Therefore the selected excipient can be used to formulate stable omeprazole tablets. This also indicates that omeprazole is compatible when mixed with all selected excipients as the concentration of omeprazole did not change significantly from day 0 to day 90.

## 4. Conclusion

The physical characteristics of omeprazole magnesium comply with US Pharmacopeia standards with regard to the fair flowability and fineness. From the above physical characteristics of omeprazole magnesium, suitable excipients with their respective amounts can be selected for the full formulation of omeprazole enteric coated tablets. Even the formulation method direct compression can be selected considering the physical characteristics of omeprazole magnesium and the excipients used.

The potential excipients, that is, sodium lauryl sulphate, lactose, Avicel PH 101, starch, water aerosil 200, and magnesium stearate, used in this study for compatibility with omeprazole magnesium were all compatible with omeprazole magnesium powder when subjected to different conditions.

However water aerosil 200 which was mixed with omeprazole magnesium showed physical changes, that is, colour changes, when subjected to all conditions showing some incompatibility with omeprazole magnesium powder. Omeprazole magnesium powder did not change when subjected to stressful conditions of higher temperatures and relative humidity.

There was no specific trend shown in moisture variation as the number of days increases and the changes were due to environment to which it was exposed; this was causing absorption changes in near infrared spectra and thus some spectra deviating from the reference spectra.

## Figures and Tables

**Table 1:** Conditions to which samples were subjected.

Container	Temperature	Humidity	Bottle status
Set A	Room temperature (30 ± 2°C)	Controlled	Plastic bottle closed
Set B	Climatic chamber (40 ± 2°C)	Relative humidity 75%	Plastic bottle opened
Set C	Oven (50°C)	Not controlled	Plastic bottle closed

**Table 2:** Composition of core tablets.

Ingredient	Specifications	Qty/tablet (mg)	Qty/batch (g)	Reasons for inclusion
(1) Omeprazole magnesium	USP	20 mg	80 g	Active
(2) SLS (SDS)	USP	2 mg	8 g	Lubricant
(3) Tablettose (lactose)	USP	76 mg	304 g	Binder
(4) Avicel ph 101	USP	60 mg	240 g	Disintegrant
(5) Starch	USP	40 mg	160 g	Diluent
(6) Water aerosil 200	USP	0.6 mg	2.4 g	Glidant
(7) Magnesium stearate	USP	1.4 mg	5.6 g	Lubricant
		200 mg	800 g	

**Table 3:** Micromeritics properties of omeprazole magnesium.

Sample	Bulk density	Tapped density	Carr's index	Hauser's ratio
Omeprazole magnesium	0.4 g/mL	0.485 g/mg	17.5%	1.2

**Table 4:** Appearance of samples in conditions A, B, and C.

	Condition A (room temperature, plastic bottle closed)						Condition B (climatic chamber, plastic bottle opened)						Condition C (oven, plastic bottle closed)					
Days	0	3	8	30	60	90	0	3	8	30	60	90	0	3	8	30	60	90
Samples																		
Omep. Mg	w	w	w	w	w	w	w	w	w	w	w	w	w	w	w	w	w	w
Omep. Mg: SLS	w	w	w	w	w	w	w	w	w	w	w	w	w	w	w	w	w	w
Omep. Mg: lactose	w	w	w	w	w	w	w	w	w	w	w	w	w	w	w	w	w	w
Omep. Mg: Avicel PH 101	w	w	w	w	w	w	w	w	w	w	w	w	w	w	w	w	w	w
Omep. Mg: starch	w	w	w	w	w	w	w	w	w	w	w	w	w	w	w	w	w	w
Omep. Mg: water aerosil	w	D.P	D.P	P	P	P	w	D.P	D.P	D.P	B	B	w	D.P	D.P	L.P	L.P	L.B
Omep. Mg: Mg stearate	w	w	w	w	w	w	w	w	w	w	w	w	w	w	w	w	w	w
Omep. Mg: all excipients	w	L.P	L.P	L.P	L.P	L.P	w	L.P	L.P	L.B	L.B	L.B	w	L.P	L.P	O.W	O.w	O.w

W: white, D.P: dark purple, L.P: light purple, L.B: light brown, and O.W: off white.

**Table 5:** Moisture content in samples in conditions A, B, and C.

Conditions	A (room temperature, plastic bottle closed)					B (climatic chamber, plastic bottle opened)					C (oven, plastic bottle closed)				
Days	3	8	30	60	90	3	8	30	60	90	3	8	30	60	90
Samples															
Omep. Mg	4.5	5.5	7.9	1.4	2.8	6.1	6.4	5.6	5.4	7	2.6	5.6	6.8	2.8	4.2
Omep. Mg: SLS	2.1	3.9	4.2	2.6	2.8	5.7	6.8	3.8	4.1	6.8	2.7	5.6	5.5	1.4	2.8
Omep. Mg: lactose	2.5	1.4	3.8	2.7	2.7	6.9	6.6	10.5	3.8	4	2.8	5.6	4.2	4.2	10.8
Omep. Mg: Avicel PH 101	3.9	5.6	4.2	4.2	2.8	6.7	6.8	8	7	6.6	2.7	5.1	5.7	4.1	5.5
Omep. Mg: starch	5.6	8.3	6.8	5.6	5.4	10.1	6.7	12.9	8.9	5.4	5.6	7.5	6.8	2.8	2.8
Omep. Mg: water aerosil	2.7	6.7	5	5.5	4.2	7.1	7	7.6	5.3	6.2	2.9	5.4	6.7	4.1	6.8
Omep. Mg: Mg stearate	1.3	4	5.2	4.2	1.4	4	4.1	9	4.2	7	3.7	2.8	8.8	4.2	4.1
Omep. Mg: all excipients	4.1	6.8	5.6	5.5	4.2	5.4	5.7	8.2	7	7.1	5.2	7.1	14.1	4.2	6.1

**Table 6:** HPLC assay results for conditions A, B, and C.

Sampled days	Omeprazole alone	Omeprazole: all excipients
Condition: A—room, uncontrolled conditions—30 ± 2°C		
Day 0	100	100
Day 7	99.97	99.11
Day 30	99.48	98.93
Day 60	99.23	98.52
Day 90	99.14	98.26

Condition: B—room, controlled conditions—40 ± 2°C/75% ± 5%RH		
Day 0	100	100
Day 7	98.65	98.23
Day 30	97.40	97.60
Day 60	97.24	97.37
Day 90	96.94	96.95

Condition: C—oven (50°C)		
Day 0	100	100
Day 7	97.73	98.39
Day 30	97.10	97.85
Day 60	96.98	97.01
Day 90	96.75	96.68
